# The Influence of Gum Arabic Admixture on the Mechanical Properties of Lime-Metakaolin Paste Used as Binder in Hemp Concrete

**DOI:** 10.3390/ma14226775

**Published:** 2021-11-10

**Authors:** Przemysław Brzyski

**Affiliations:** Faculty of Civil Engineering and Architecture, Lublin University of Technology, 40 Nadbystrzycka Str., 20-618 Lublin, Poland; p.brzyski@pollub.pl; Tel.: +48-81-538-4448

**Keywords:** lime, metakaolin, gum arabic, mechanical parameters, pore size distribution, hemp concrete

## Abstract

Organic admixtures based on polysaccharides are used in construction for modifying the properties of mortars and concretes. Gum arabic is an example of a polysaccharide-based biopolymer. The aim of the article was to investigate the possibilities of improving the strength parameters of a binder paste based on hydrated lime and metakaolin. The paste was modified with powdered gum arabic at 1%, 3% and 5% (by mass) as a partial replacement for the binder mix. The influence of the admixture on the pore size distribution as well as flexural and compressive strength was investigated. The admixture enhanced the total porosity of the paste, increasing the pore diameter compared with the reference formulation. The increase in porosity, in turn, did not reduce the mechanical strength. Conversely, the admixture in the amount of 3% and 5% caused a significant increase in the flexural (by about 300% in relation to reference paste) and compressive strengths (by 25% and 60%, respectively). The tested pastes were used as a binder in a composite based on hemp shives. The influence of binder modification on the water absorption and compressive strength of hemp concrete was tested. The strength of the composite soaked in water was also tested. The modification of the binder with gum arabic in the amount of 3% and 5% increased the compressive strength of hemp concrete (not soaked in water) by 53% and 92%, respectively and reduced the mass absorptivity by 6.6% and 10.4%, respectively.

## 1. Introduction

Hemp concrete (hemp-lime composite) is mainly used as insulation filling in timber frame wall structures. It consists of hemp shives and a binder based on hydrated lime. This type of lime is also used to produce mortars or other composites, presently this is mostly in a powdered form but in the past was primarily in the form of lime putty [1]. This binder hardens very slowly in the carbonation process, i.e., calcium hydroxide binds carbon dioxide in the air in the presence of moisture. The effectiveness of the carbonation process depends on the air temperature, relative humidity, and CO_2_ concentration in the atmosphere [2]. It can take a long time to completely convert the calcium hydroxide into calcite in the mortar [3]. Due to the slow setting and hardening process of lime, for practical reasons, this binder is modified with additives or admixtures that speed up setting, improve strength, and enhance frost and water resistance.

One way to modify hemp concrete is to partially replace lime with pozzolana. Pozzolans such as zeolite [4,5], fly ash [6], wood ash [7], heat treated clays [1] or silica fume [8] have been used for modifying lime mortars or other composites based on a lime binder. In this study, metakaolin was used as pozzolanic material. It is often employed as a partial replacement of lime pastes or mortars, which is confirmed by numerous scientific publications [5,9,10]. Metakaolin is obtained by calcining kaolin clay and then grinding the calcined product. The main components in the chemical composition of metakaolin are SiO_2_ (about 40–50 wt.%) and Al_2_O_3_ (about 30–40 wt.%) [5,11]. In lime-metakaolin mixtures, these components react in the presence of water with a portion of calcium hydroxide to form calcium silicate hydrates as well as calcium silicate and aluminate hydrates. The remainder of the calcium hydroxide reacts with carbon dioxide from the atmosphere in the carbonation process to form calcium carbonate. Modification of lime pastes or mortars with metakaolin improves the parameters of these materials. Gameiro et al. [12] showed that lime mortar containing 30% metakaolin (with a binder:sand weight ratio of 1:4) exhibited compressive strength after 90 days of maturation which was about a 14-fold increase over the mortar without metakaolin.

In ancient times, organic additives and admixtures were used to improve the properties of lime mortars. Currently, many research works are trying to recreate old mortar recipes and examine their properties. Zhao et al. [13] used sticky rice, tung oil and pig blood as additives. He proved that rice slurry at 5 wt.% accelerates setting and hardening as well as increases compressive strength, tung oil at 5 wt.% strongly improves water resistance, while pig blood accelerates setting and hardening, and enhances water resistance. Ventola et al. [14] showed that the addition of animal glue increased the mechanical strength of the lime mortar twice and that the use of olive oil as additive decreased the pore size and reduced their volume. In turn, Shi et al. [15] discovered, i.a., proteins and starch in the composition of ancient lime mortars. Lopez et al. [16] used a solution of sugar and yeast, which released CO_2_, as an additive that accelerated carbonation. Organic additives are susceptible to the growth of microorganisms that cause their decomposition. However, historic buildings around the world prove that mortars with organic additives can survive for hundreds of years [15]. The reason may be that the carbonation process is very slow and calcium hydroxide in the deeper areas of historical mortars still has not transformed into calcite, thus leaving a strongly alkaline environment, which is destructive for microorganisms [17].

Currently, experiments with the addition of natural polymers based on polysaccharides for building materials are also being carried out. Examples of these polymers are guar gum, welan gum, gum arabic, and sticky rice. Vysvaril et al. [18] proved that the admixture of hydroxypropyl guar gum in the amount of 0.05–1% by weight of lime improves water retention in lime mortars. In turn, Izaguirre et al. [19] showed—based on a compressive strength test—that the admixture of this polysaccharide improves the frost resistance of lime-based mortars. Mbugua [20] investigated the influence of gum arabic (0.3–1.1% dosage) on the properties of cement mortar. He proved that the admixture increases the spread of the mortar and that the compressive strength decreases with the increase in the gum arabic content, while maintaining the same w/c ratio. However, the use of gum arabic allowed a reduction in the amount of water, which in turn increased the strength. Other studies [21] examined the effect of gum arabic admixtures on the properties of concrete. It was found that the air content in the concrete increased along with the admixture content, which resulted in a decrease in compressive strength. The admixture above 2% caused the mixture to liquefy considerably, but due to the adhesive properties of the gum, this fluidity was quickly lost. This property can be used in the production of prefabricated elements. The admixture also allowed for a significant reduction in the amount of water in the concrete mix recipe. Zhang [22] used welan gum as an admixture in the cement mortar and proved that the admixture in the amount of 0.1% in relation to the cement mass significantly reduced the fluidity of the mortar immediately after mixing. Izaguirre et al. [19] used guar gum in the amount of 0.3% of the binder weight as a water-retaining admixture in the lime mortar. This biopolymer improved the durability of the mortars under freezing and thawing conditions.

A lot of research work focuses on the amylopectin admixture, i.e., the polysaccharide contained in rice [13,23,24,25,26]. Yang et al. [23] stated that the admixture of sticky rice in the amount of 3% significantly improved the mechanical strength of lime mortars. Researchers proved that amylopectin inhibits the growth of calcium carbonate crystals, which creates a denser mortar microstructure composed of small calcium carbonate crystals—the result is greater strength [13,24,25,26]. In addition, sticky rice covers calcium carbonate crystals and aggregate, which makes the mortar more resistant to weather conditions and water [24,25]. Ventola et al. [14], in turn, claim that the addition of polysaccharides to lime mortars accelerates carbonation by releasing CO_2_ during the fermentation of these organic additives. Pradeep and Selvaraj [27] tested lime mortars modified with fermented herbal extract containing about 80–85% polysaccharides in its composition. The compressive and flexural strengths of modified lime mortars increased by 37% and 34% respectively, in comparison with the reference mortars.

The high water absorption of shives is a problem in the production of hemp concretes [28,29]. This requires the use of large amounts of water to be able to mix the ingredients and obtain the right consistency. The result is an extended drying time for the material. It is difficult to establish the correct amount of water so that the shives do not absorb some of the water needed to set the binder properly. Since admixtures of polysaccharide-based biopolymers are used as water-retaining agents in mortars and concretes, in this study gum arabic was used for this purpose. Additionally, it was anticipated that the sticky consistency of the dissolved gum arabic solution can improve the adhesion of the binder to the shives, thus positively influencing the mechanical strength of the hemp concrete. Hemp-lime composites are characterized by a compressive strength of around 0.1–0.8 MPa [29,30,31,32,33,34]. Strength is affected by, among other things, the type of binder [30,31,33]. There is a need to search for new binder components that would improve strength, and thus extend the scope of application of composites.

As mentioned in the previous paragraphs, there are several papers describing the research on the modification of mortars or concretes with organic admixtures based on polysaccharides. This article will help broaden the knowledge of their influence on selected properties of building binders. The paper discusses modification of lime-metakaolin paste with a gum arabic admixture. This substance is commonly used in the food and pharmaceutical industries as a thickening agent. It is also employed for the production of adhesives and artistic paints. The influence of the admixture and its variable amount on the pore size distribution as well as mechanical parameters (flexural and compressive strength) of the paste was tested. The main aim of the article was to assess the possibility of using the examined pastes as a binder in a composite based on hemp shives. The influence of the applied binders was determined on the basis of the results of compressive strength tests. Furthermore, improving the strength of a lime binder after modification with gum arabic could broaden the area of application of this binder.

## 2. Materials and Methods

### 2.1. Mix Design

**Figure 1:**
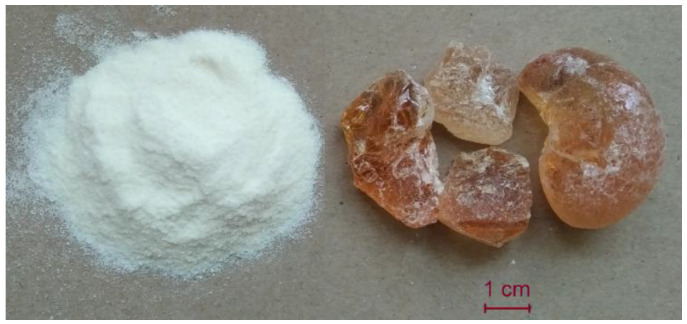
Gum arabic in the form of powder and crystals.

The binder was based on hydrated lime CL-90s. Metakaolinite was used as a partial replacement for lime. The paste reference formulation contained 90% hydrated lime and 10% metakaolin (based on the total weight of the binder). Metakaolin was used because it was expected that if hydrated lime was used alone, the paste samples would be too brittle, with poor strength and wide dispersion of results, making it difficult to draw conclusions. Although larger amounts of metakaolin were applied in other works [9,28], it was decided to use the lesser amount, expecting that the admixture of gum arabic will provide an additional increase in strength. The water to binder ratio was assumed to be 0.65 and constant for all recipes. The admixture of gum arabic was 1%, 3% and 5% (by mass) as a partial replacement of lime-metakaolin mix. Gum arabic is a natural resin (biopolymer), a multi-molecular carbohydrate obtained from Senegalese acacia and contains about 80–84% polysaccharides. Chemically, it is a mixture of galactose (35% to 45%), arabinose (25% to 45%), glucuronic acid (6% to 19%), rhamnose (4% to 13%), and also mineral salts (sodium, magnesium, potassium, calcium) [35]. In the investigation, it was used in the form of a powder, but this material is also available in the form of solid pieces (Figure 1). Such crystals can be obtained by mixing the powder with a small amount of water and then drying the solution. This natural resin in the sticky form hardens under the influence of air. The water solubility of gum arabic is about 500 g/dm^3^ and the pH is 5 [36].

The recipe symbols and their composition are presented in Table 1. The number in the lime-metakaolin paste (LM) symbol means the percentage of gum arabic (GA) in relation to the binder weight (hydrated lime + metakaolin).

X-ray fluorescence (XRF) (Panalytical, Eindhoven, The Netherlands) was used to determine the composition of the tested pastes. Gum arabic was applied as a partial substitute for the lime and metakaolin mixture. Therefore, the amounts of individual chemical components in the composition clearly decreased with the increasing gum arabic content. Trace amounts of K_2_O have been reported in the mixtures with biopolymer. The ingredients present in trace amounts remain constant regardless of the recipe. Table 2 presents the chemical composition of the tested paste mixtures. Organic components were included in the loss on ignition.

**Figure 2:**
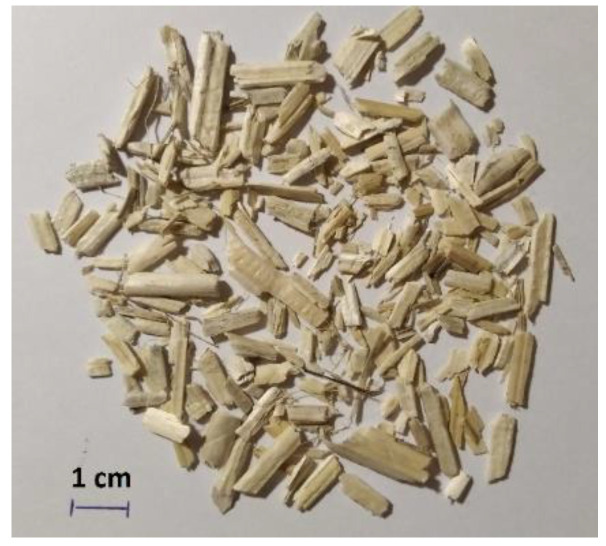
Hemp shives used in studies.

**Figure 3:**
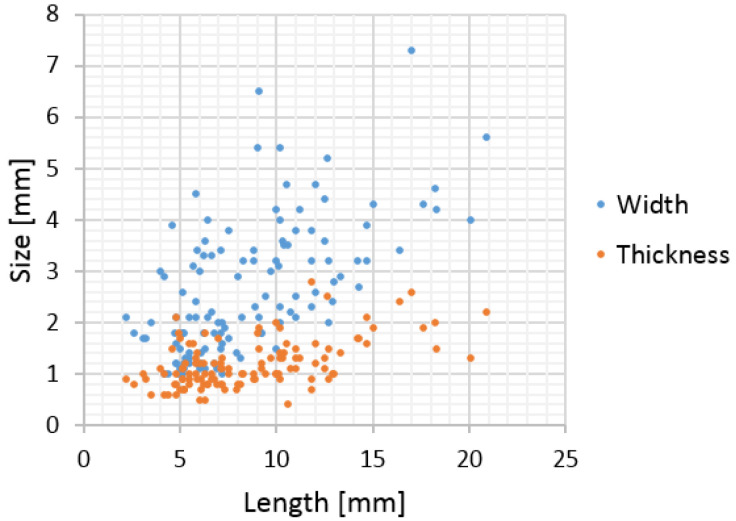
Hemp shives granulation.

The developed pastes were used as binder in the composites based on the hemp shives obtained from the Polish variety named Białobrzeskie and produced by the Polish company Podlaskie Konopie (Białystok, Poland). Shives are characterized by a bulk density of about 120 kg/m^3^, a total porosity of about 85% and high water absorption. During the short period of immersion in water, they are able to absorb the amount of water equivalent to three times their weight. The hemp shives used in studies are shown in Figure 2, while their granulation is shown in Figure 3. The most common length of the shives in the mixture were 5–10 mm; width in the range of 1–4 mm and thickness in the range of 0.5–1.5 mm. However, there were also many shives with a length of more than 10 mm. The mixture is varied, which is advantageous in order to obtain the proper compaction and structure of the composite.

The hemp-lime mixture symbols and their composition are presented in Table 3. The number in the hemp-lime concrete (HL) symbol means the percentage of gum arabic (GA) in relation to the binder weight (hydrated lime + metakaolin). The adopted weight ratio of shives to binder was also used in other works [28,30,37,38]. As assumed in the introduction, the admixture of gum arabic in the binder made it possible to reduce the amount of water in the hemp-lime mixture. The mixtures containing the LM-3GA and LM-5GA binder, despite the lower water content, showed a suitable, sticky consistency.

### 2.2. Research Scope and Program

The research was divided into two parts. The first part concerned the binder used in the considered hemp-lime composites. Strength tests (flexural and compressive strength) were performed; in addition, the chemical composition of the binders, pore distribution and SEM images of binder paste structure were analyzed.

The second part concerned the compressive strength tests of hemp-lime composites performed on the basis of the previously tested binders. Characteristics of the composite under axial load were checked in three cases:

1. composites cured under natural conditions for 90 days (marked as HL-GA),
2. composites cured under natural conditions for 90 days, and then immersed in water for 48 h (marked as HL-GA soaked),
3. composite cured under natural conditions for 90 days, then immersed in water for a period of 48 h, and subsequently naturally dried for 10 days (marked as HL-GA dried).

Checking the impact of changes in strength parameters due to water saturation is related to the fact that the composite absorbs large amounts of water and consists of components with low water resistance. The mass absorptivity test of the composites was also performed.

### 2.3. Preparation of Specimens

#### 2.3.1. Binder

Gum arabic was added to the measured amount of water, according to the recipes presented in the table. Stirring with a manual mechanical mixer was performed until the gum arabic dissolved and a homogeneous solution was obtained (mixing time was about 2 min). When mixing at high speed, foam was formed on the surface of the solution, becoming more intense as the admixture content increases. The dry components of the binder, i.e., hydrated lime and metakaolin, were premixed by hand and then added to the solution of water with gum arabic. All ingredients were mixed until the paste was homogeneous (about 2 min). The mixture with 3% and 5% gum arabic became visibly liquefied. The foam, formed in a solution of water and gum arabic, caused air bubbles to be introduced into the mixture, making it more fluid. This phenomenon was also observed in [21]. The admixture of gum arabic in the amount of 3% and 5% liquefied the binder mixture; however, the amount of water was not reduced in these recipes because the equal water-to-binder ratio was assumed for a more accurate assessment of the effect of the admixture on the properties.

The mixture was placed in three-part molds with dimensions of 40 mm × 40 mm × 160 mm and compacted for 15 s on a vibrating table. The samples were matured under air-dry conditions (temperature: 21 °C ± 2 °C and relative humidity: 50% ± 5%) for 90 days. All tests were performed after this maturation period.

#### 2.3.2. Hemp-Lime Concrete

First, the hemp shives were mixed with part of the water (half of the total amount). The dry blended binder components (hydrated lime and metakaolinite) were then added, and all components were mixed. Afterwards, 1/4 of the amount of water in which the gum arabic was dissolved was added. Finally, the remainder of the water was added and mixed until the desired consistency was obtained (viscous, enabling to form a compact ball of the mixture by hand). The mixture was placed in molds with dimensions of 100 mm × 100 mm × 100 mm and compacted by hand using a wooden compactor with the cross-sectional dimensions of 20 mm × 30 mm. The same method was used in [31]. In other studies [29], the mix was compacted using a hydraulic press. In order to obtain comparable volume densities of the samples, the mass of the mold with the samples was controlled during the molding process, aiming at the most similar mass. A similar procedure was used in other studies [30]. The samples were matured under air-dry conditions (temperature: 21 °C ± 2 °C and relative humidity: 50% ± 5%) for 90 days. All tests were performed after this maturation period.

### 2.4. Binder Testing

#### 2.4.1. Pore Size Distribution

The principle of the mercury porosimetry method is to determine the amount of mercury that is forced into the pores of the tested material, assuming that the increase in pressure will fill progressively smaller pores. The measuring range for the above-mentioned method covers pores with a size of about 0.003 to 360 μm. The porosity of the four pastes was determined on an Autopore IV 9510 mercury porosimeter (Micromeritics, Norcross, GA, USA). In order to remove the physically absorbed water vapor and other gases from the surface, the samples weighing ~0.6 g were dried at 105 °C before the actual measurement. The equivalent pore radius was determined using Washburn’s Equation (1). Distribution of pore size and surface area was presented by means of cumulative and differential curves in the diameter range from 0.003 µm to 360 µm. The mean pore diameter (D) was obtained assuming that all pores are cylindrical; thus, when the total pore volume (V = πr^2^L) is divided by the total pore area (S = 2πrL) the value of the mean pore diameter is 4 V/S. The bulk density of the samples was determined according to Formula (2). The apparent density of the samples was determined in line with Formula (3).

(1) $$R=\frac{2\cdot \sigma_{m}\cdot \cos\theta_{m}}{P_{m}}$$

where *R* is the pore radius, *σ_m_* is the mercury surface tension (0.485 J/m^2^), *θ_m_* is the mercury contact angle (assumed 130°), and *P_m_* is the external pressure (Pa).

(2) $$d_{n}=d_{Hg}\cdot M/\left(M1-M2+M\right)$$

where *d_n_* is the bulk density (g/cm^3^), *d_Hg_* is the density of mercury at the measurement temperature (g/cm^3^); *M* is the sample mass (g), *M*1 is the mass of mercury-filled dilatometer (g), and *M*2 is the mass of the mercury-filled dilatometer and sample (g).

(3) $$d_{p}=1/\left(\frac{1}{d_{n}}-V\right)$$

where *d_p_* is the apparent density (g/cm^3^), *d_n_* is the bulk density (g/cm^3^), and *V* is the total pore volume (cm^3^/g). 

The total porosity of the samples was calculated according to Formula (4).

(4) $$P=V\cdot d_{n}\cdot 100\%$$

In order to analyze the pore distribution in more detail, pictures were taken with a Quanta 250 FEG scanning electron microscope (FEI, Hillsboro, OR, USA). Samples of binders with an uncut fracture surface taken from the samples intended for strength tests were glued to the carbon holder with carbon glue. The samples prepared in this way were coated by sputtering with a carbon layer with a thickness of about 50 nm in order to obtain conductivity on the sample surface.

#### 2.4.2. Flexural and Compressive Strengths

The flexural strength was tested in accordance with the PN-EN 1015-11 standard using an MTS 809 hydraulic press (MTS System Corporation, Eden Prairie, MN, USA) on 40 mm × 40 mm × 160 mm samples. Five samples from each recipe were used. The head displacement was assumed to be 0.2 mm/min. The compressive strength was tested on the basis of the PN-EN 1015-11 standard on the halves of 40 mm × 40 mm × 160 mm samples obtained in the flexural strength test. Eight samples from each recipe were tested. The displacement of the compression head was assumed to be 3 mm/min. In other studies [39], a slower displacement increment of 0.7 mm/min was assumed. In other studies [13], the load increment was controlled at the level of 0.02 MPa/s. The test was carried out after 90 days of maturing the samples under air-dry conditions.

A phenolphthalein test was performed to assess whether the modification of the binder with the biopolymer influenced the progress of carbonation. The test was performed on the surface where the break of the sample occurred. The samples were sprayed with 1% phenolphthalein in an alcohol indicator solution. Carbonation is the process of setting and hardening the lime binder and affects the mechanical strength.

### 2.5. Hemp-Lime Concrete Testing

#### 2.5.1. Mass Absorptivity

The water absorption test (mass absorptivity) consisted in immersing the samples with dimensions of 50 mm × 60 mm × 120 mm in water and measuring the weight gain at specified intervals of time. Due to the high water absorption of the hemp-lime composite in the first seconds after immersion in water, which is confirmed by tests [28], it was decided to increase the number of readouts during the initial immersion period. The first measurement was conducted after 5 s of immersion. The periods for reading the mass are: 5 s, 15 s, 30 s, 1 min, 15 min, 30 min, 1 h, 3 h, 12 h, 1 d, 2 d, 3 d, 5 d, and 6 d. Four samples from each recipe were used for the tests.

#### 2.5.2. Compressive Strength and Young’s Modulus

There are no standards for testing the compressive strength of hemp-lime composites or similar materials based on ingredients of plant origin. The hydraulic compression press, with a load range of 0–250 kN, was set individually. The displacement increment of the compression head was set at 5 mm/min. The increase in the displacement of the head was also individually selected by other researchers: 0.2 mm/min [32], 3 mm/min [40,41,42], 5 mm/min [29,31,34,40]. The dimensions of the tested samples are 100 mm × 100 mm × 100 mm. The same dimensions were adopted in [30]. In the literature, there are different dimensions of samples for compressive strength tests. Cylindrical [29,32] as well as cuboid-shaped [40,42,43] samples are also used.

Composites were tested after 90 days of maturation in the following amounts:

- 4 samples from each mixture, not subjected to water treatment,
- 3 samples of each mixture, soaked in water for 48 h,
- 3 samples of each mixture, removed from water after soaking for 48 h and then dried under natural conditions for 10 days.

The Young’s modulus of composites was determined from the maximum slope of the curve in the initial phase, in which the material behaved elastically. The same method has been used or described in other studies of hemp-lime composites [29,30,32].

## 3. Results

### 3.1. Binder

#### 3.1.1. Pore Size Distribution

Table 4 shows the average results of the paste tests performed by means of mercury porosimetry.

**Figure 4:**
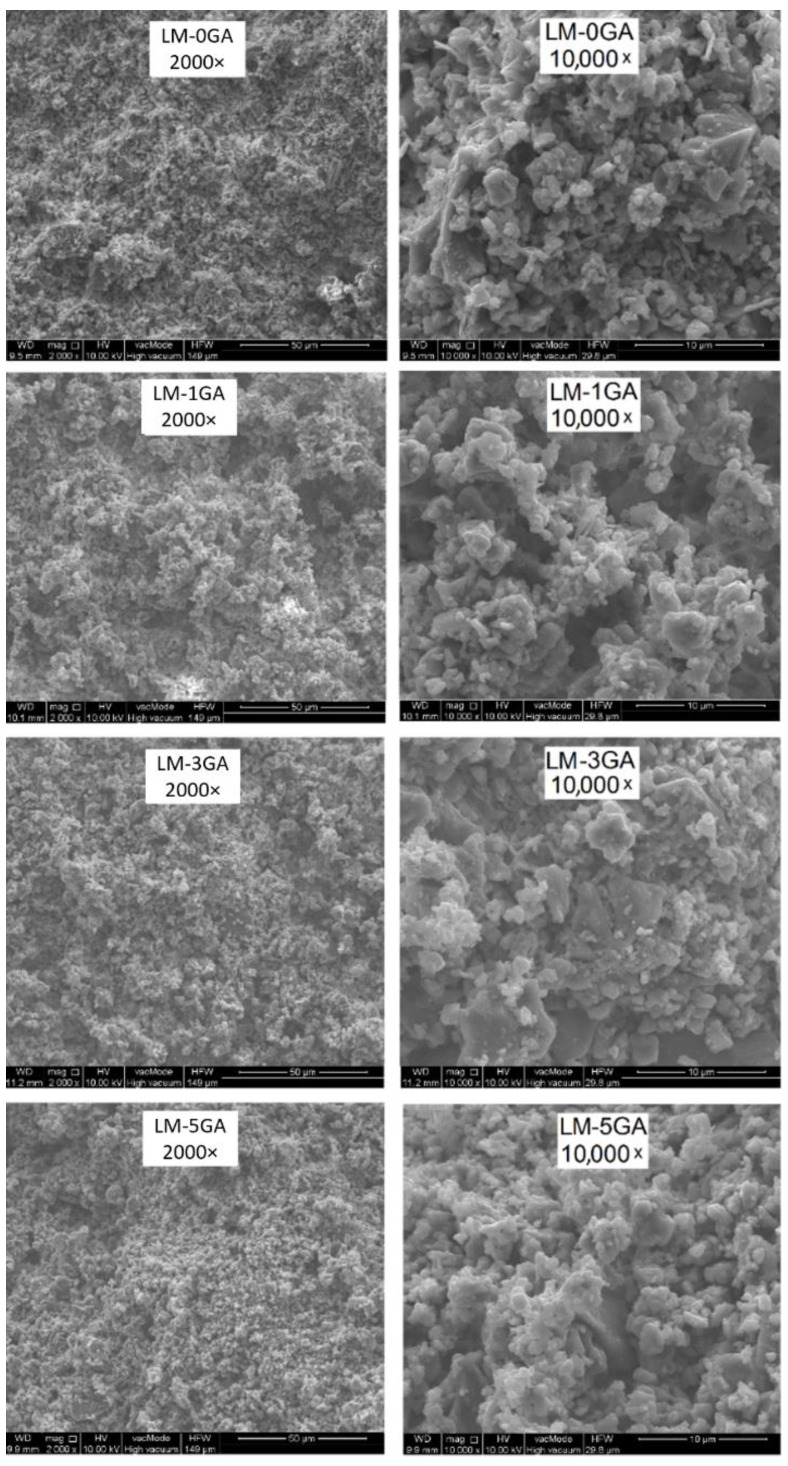
SEM images of paste samples.

The structure of the pastes is shown in the photos taken with a scanning microscope (Figure 4).

**Figure 5:**
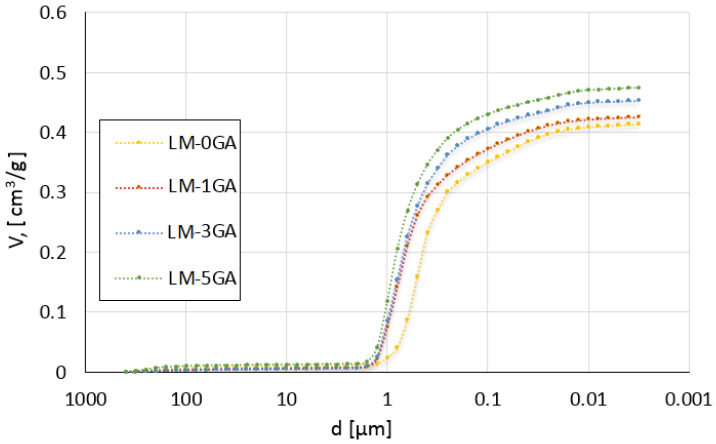
Cumulative volume of intruded mercury versus pore diameter for the tested lime-metakaolin pastes.

**Figure 6:**
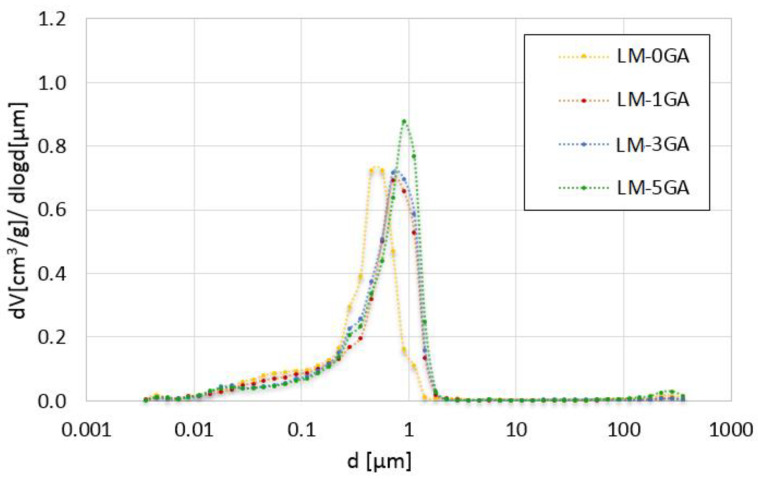
Differential volume of intruded mercury versus pore diameter for the tested lime-metakaolin pastes.

**Figure 7:**
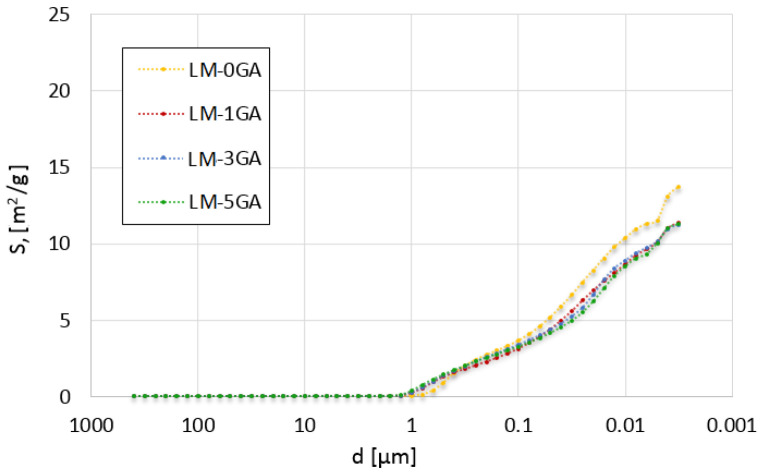
Total pore surface versus pore diameter for the tested lime-metakaolin pastes.

The cumulative intrusion versus pore diameter curves and differential curves is shown in Figure 5 and Figure 6, respectively. The total pore surface versus pore diameter curves is shown in Figure 7.

#### 3.1.2. Flexural and Compressive Strengths

**Figure 8:**
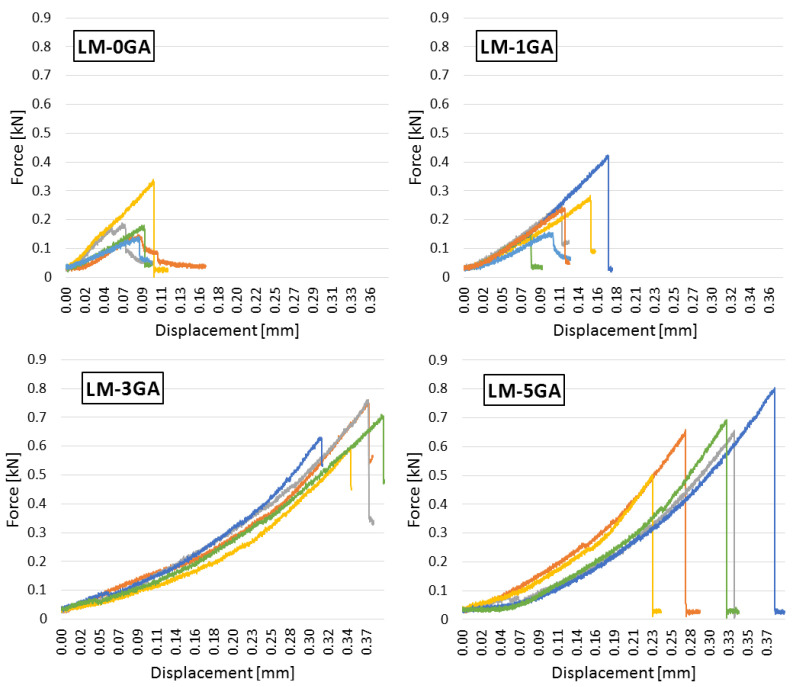
The dependence of the bending force on the displacement of the press head.

The relationship between the bending force and the displacement of the press head of all samples within an individual recipe is shown in Figure 8.

**Figure 9:**
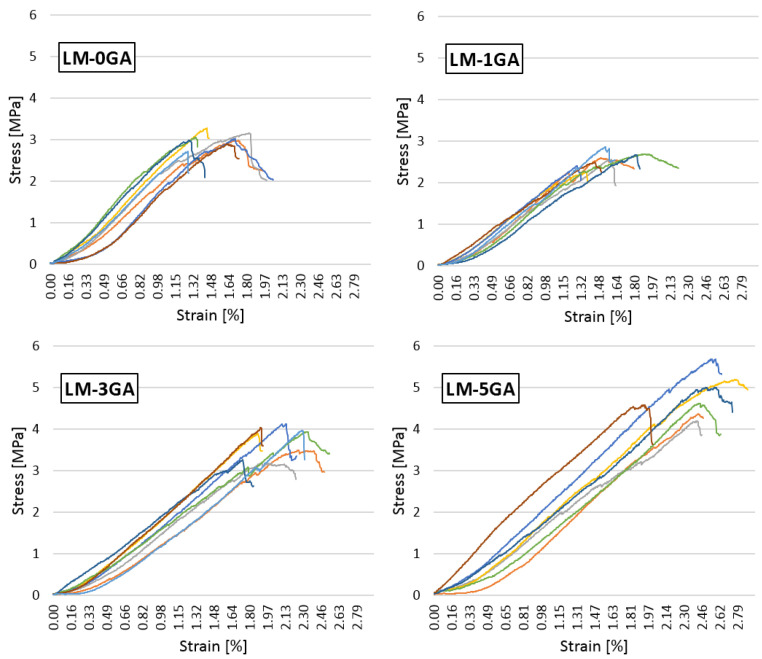
The dependence of the stress on the strain.

The relationship between the stress and the strain of all samples within an individual recipe is shown in Figure 9.

**Figure 10:**
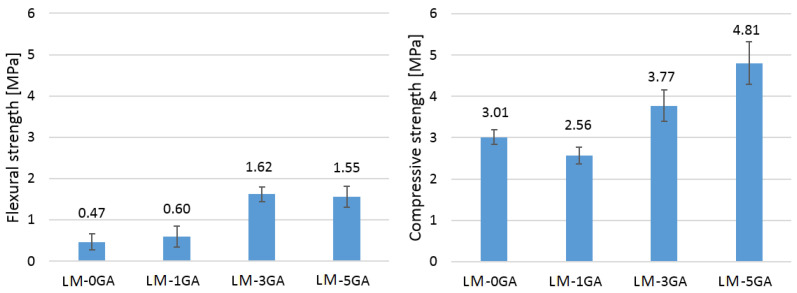
Average values of flexural strength (on the **left**) and compressive strength (on the **right**) of the tested samples (error bars mean standard deviation).

The averaged values of flexural and compressive strengths are shown in Figure 10.

**Figure 11:**
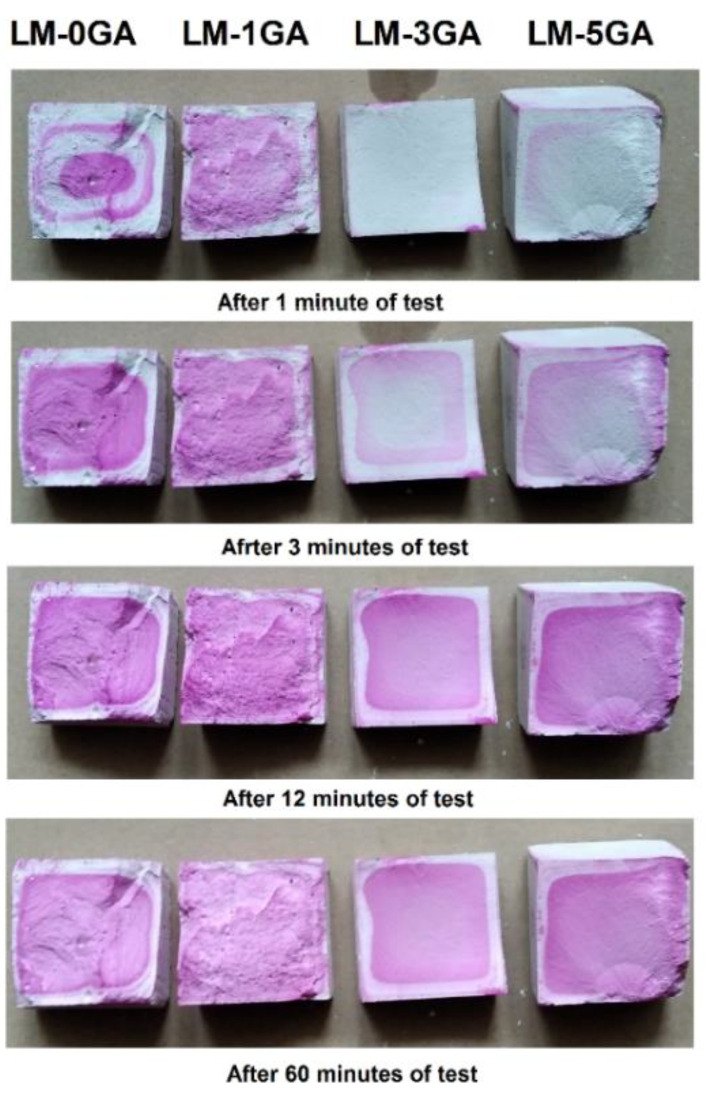
Progress of carbonation of the tested pastes.

Figure 11 shows the observations during the phenolphthalein test.

### 3.2. Hemp-Lime Composites

One of the main parameters of a building material influencing its other properties is the bulk density. It affects the water absorption and strength of the material. Table 5 shows the bulk density of hemp concretes and the standard deviation. All recipes use the same proportion of binder to shives. The differences in densities result from the compaction process.

#### 3.2.1. Mass Absorptivity

**Figure 12:**
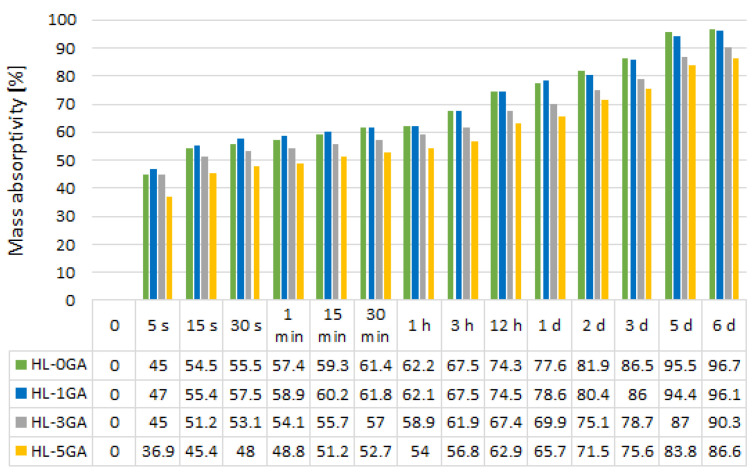
Average values of mass absorptivity of the tested composites.

The averaged values of mass absorptivity are shown in Figure 12.

#### 3.2.2. Compressive Strength and Young’s Modulus

**Figure 13:**
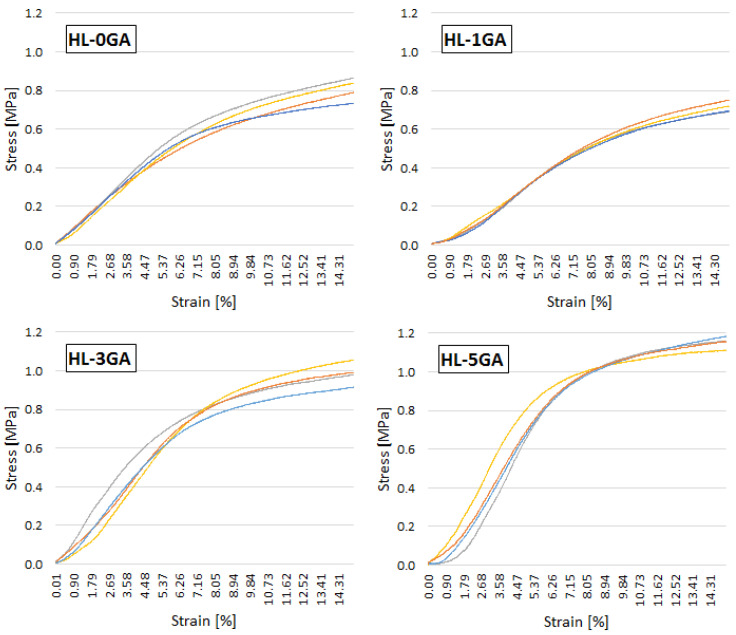
Stress–strain relationship of tested hemp-lime composites.

The stress-strain curves of tested hemp-lime composites of all samples within an individual recipe are shown in Figure 13.

**Figure 14:**
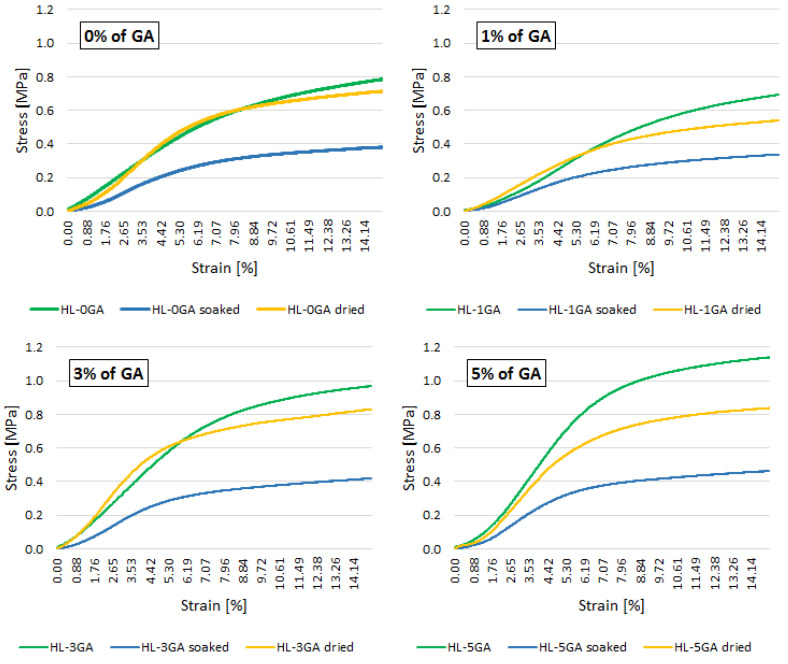
Stress–strain relationship of tested composites in various variants (average values).

Figure 14 shows the stress–strain curves of the hemp-lime samples tested in various variants. Average values are given in the graphs.

**Figure 15:**
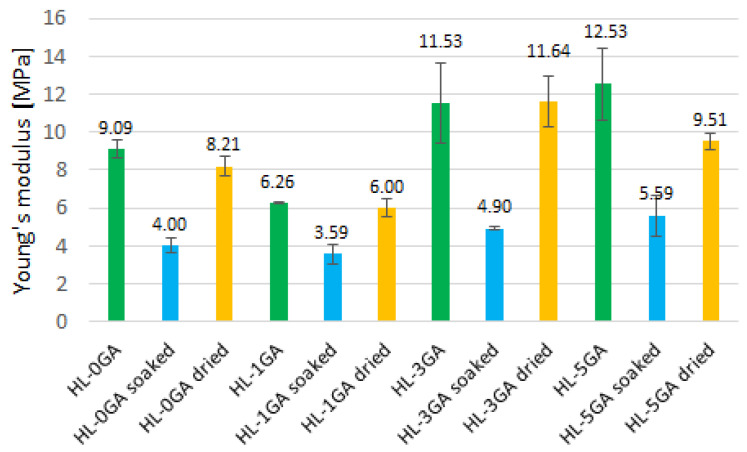
Young’s modulus of the composites (error bars = standard deviation).

The results of Young’s modulus are presented in the diagram (Figure 15).

## 4. Discussion

### 4.1. Binder

#### 4.1.1. Pore Size Distribution

Porosity is an important characteristic of the microstructure of building binders. This feature influences other relevant physical and mechanical parameters of materials; the total pore volume, size and distribution are important as well [44]. As the admixture increases, the binder density decreases. This is due to the increase in the total pore volume as a result of the air bubbles introduced during mixing. Similar observations were made in the case of concrete modified with gum arabic [21].

The total porosity of the paste increases along with the content of gum arabic. It is related to the liquefaction of the paste mixture during mixing, due to the addition of gum arabic mixed in water (dissolved). The gum arabic admixture in the amount of 3% and 5% acts as an air-entraining agent that entraps tiny air bubbles during the paste mixing process. The difference between the total porosity of the reference paste and the recipe with the 1% gum arabic admixture is small because the amount of admixture was too little to liquefy the mixture (when gum arabic was mixed with water, the intensity of air bubbles was much lower).

The average pore diameter also increased with the admixture content, and thus the specific pore surface area decreased significantly. The admixture caused a significant increase in average diameter (by about 25–40% compared with the reference paste). 

The SEM photos (Figure 4) show the breakthrough of the samples. Due to the relatively short maturation time of the samples (90 days), the carbonation process was probably advanced only in the surface layers of the samples; therefore, the material taken from the middle areas of the samples was selected as representative for both porosity tests and SEM analysis. The SEM images do not show distinct differences among the microstructure of the reference and modified pastes. The structure of the reference sample is the most compact, the pore distribution is even, and no significant difference in pore size can be seen. The structure of samples containing higher amounts of gum arabic (3% and 5%) is more heterogeneous, larger pores are visible. Gum arabic was dissolved in water, and the solution of GA and water was homogeneously distributed as a film over the binder grains, though this is hard to see with the SEM images. The creation of a film by the admixture of polysaccharides is also confirmed by other studies [22,45].

All pastes have a similar pore size distribution in the case of cumulative pore volume, mainly composed of pores with a diameter between 0.1 µm and 1 µm (Figure 5). This is the typical range of pore diameters for lime pastes [46]. There is a noticeable difference in the pore size distribution between the samples with the admixture and the reference sample. The admixture of gum arabic increased the content of pores with a diameter ≥ 1 µm. In the case of the reference paste, their volume is about 0.02 cm^3^/g, and in the case of modified pastes, it is about 0.08–0.12 cm^3^/g. The greatest differences in the volume of pores can be seen in the range of <1 µm and ≥0.6 µm. In the case of the reference paste, their volume is 0.06 cm^3^/g, and in the case of modified pastes, 0.14–0.15 cm^3^/g. The greater pore volume of the aforementioned average sizes may be related to the mixing procedure and the introduction of air bubbles that were formed by mixing gum arabic in water. In turn, other researchers [47] have stated that the pores with a diameter greater than 1 µm can be considered as formed as a result of the mixing process and the introduction of air into the mixture. The content of pores with a diameter of 0.01–0.1 µm was 0.06 cm^3^/g in the reference paste and 0.04–0.05 cm^3^/g in the modified pastes. Generally, with the decrease of the pore diameter, the differences in their volume in the paste samples decrease and stabilize. In each range of pore sizes, their total volume increases along with the admixture of gum arabic. The opposite is true for the pores smaller than 0.1 µm—in this case, their volume is larger in the reference sample, and decreases with the increasing admixture content. This may indicate that the admixture of gum arabic does not increase the number of pores in the whole range of diameters, only in selected range. However, the gum arabic admixture increases their diameter, which leads to greater total pore volume in the paste.

Figure 6 shows that the reference paste contains significantly more pores with a diameter of 0.3–0.6 µm and slightly more pores with a diameter below 0.3 µm than those containing gum arabic. The greatest difference can be seen in the number of pores with a diameter of about 0.5 µm. Otero et al. [45] has confirmed that the admixture of another polysaccharide (found in sticky rice) and nanolime also slightly reduced the number of pores in the diameter range 0.01–0.3 µm in the lime mortar. The samples with gum arabic have a similar pore size distribution in the case of differential volume. However, the 5% admixture caused a significant increase in the number of pores in the diameter of about 1 µm, compared with the samples with the lower admixture content. However, each amount of admixture caused a significant increase in the number of pores with a diameter above 0.7 µm, compared to the reference paste. The greatest difference is visible in the range of 0.9–1.4 µm diameters. Similar observations were shown in [19], where the modification of lime mortar with guar gum caused a significant increase in the content of pores with a diameter of 0.9–2 μm. In this work, the pores with a diameter above 1.4 µm are practically non-existent in the reference sample. On the other hand, the maximum pore diameter that occurs significantly in the pastes containing gum arabic is about 1.8 µm. In other works [19,45], after modifying the lime mortars with polysaccharides (sticky rice, guar gum), there was a significant decrease in the content of pores with a diameter above 2 µm. There are practically no pores of such mean diameters in the pastes tested in this work, however, in the presented literature [19,45], lime mortars were examined, not pastes. In addition, other polysaccharides that may have a different effect were used, and different ratios of water to binder were also used. When the gum arabic was mixed (dissolved) in water, a foam was formed with large bubbles visible, however, when introduced into the paste mixture, mixed and compacted, the larger bubbles could be destroyed.

The relationship between the total content of pores, in a given range of diameters presented in Figure 7, and their total surface can be observed. The admixture of gum arabic caused a reduction in the total surface of pores with a diameter of less than 0.3 µm. In turn, the total surface of pores greater than 0.3 µm is larger in the reference paste than in the modified ones. The differences between the pore surface values in binders containing different amounts of admixture are slight. The difference can only be seen in the pore diameter range of 0.02–0.05 µm, where the pore surface decreases with the increasing admixture content. This observation is explained in Figure 6, where it can be noticed that the number of pores in this mean range decreases with the increasing content of gum arabic.

#### 4.1.2. Flexural and Compressive Strength

**Figure 16:**
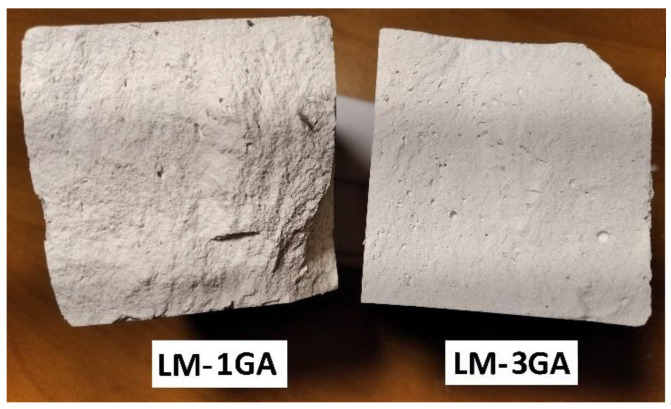
Fracture surface of the samples after the flexural strength test.

The admixture of gum arabic significantly enhances the flexural strength. The strength increases along with the admixture content. The most similar values of the destructive force, as well as the forms of graphs, can be observed in the case of the samples with an admixture of 3% (Figure 8). The paste prepared according to the LM-3GA recipe was characterized by the most regular and compact structure, which could be seen on the fracture surface of the samples after the test (compared with the LM-1GA samples, Figure 16). All samples from the LM-3GA recipe were broken with a deflection of similar values, 0.30–0.39 mm. The paste prepared according to the LM-5GA recipe, despite the highest strength, was characterized by a large spread of the maximum deflection. The collapse occurred with a deflection of 0.23–0.39 mm (Figure 8).

In the case of flexural strength, a significant influence of the admixture of gum arabic in the amount of 3% and 5% on the strength value is noticeable. The admixture in the amount of 1% turned out to be too small to effectively cover the lime particles characterized by a large specific surface. It may also be important that the admixture in larger amounts (3% and 5%) caused the mixture to liquefy, so that the lime particles were more thoroughly covered by the coating of the gum arabic solution. The result was an approximately threefold increase in the flexural strength (Figure 10).

The modification of the lime-pozzolanic paste with gum arabic influenced the compressive strength. The admixture in the amount of 1% slightly decreased the strength, while the higher amounts of the admixture (3% and 5%) significantly improved the compressive strength. There are no significant deviations in the behavior of the individual samples under increasing load (Figure 9). The most similar behavior of the samples can be seen in the case of the paste formulation with 1% gum arabic. Curves are inclined to the horizontal axis at a similar angle regardless of the recipe. It can therefore be concluded that the admixture of gum arabic does not affect the stiffness of the paste.

In the case of the compressive strength, the admixture of 1% resulted in a negative effect. The result was a reduction in strength by about 15%. The mixture LM-1GA was thick (less workable than the reference recipe) and less homogenized and this could be the reason for the lower strength than LM-0GA. In turn, the admixture in the amount of 3% and 5% improved the compressive strength of the paste by about 25% and 60%, respectively (Figure 10). Gum arabic dissolved in water exhibits sticking properties, so this physical phenomenon could have increased the strength by bonding the lime particles together. The results presented are very satisfactory, taking into account the fact that most research works prove that organic additives usually lower or slightly improve the strength parameters of pastes, mortars or concretes. The authors speculate that the effects would be even better if the water-to-binder ratio was reduced. It was possible in the LM-3GA and LM-5GA recipes, but the assumption was the only variable in the form of the amount of admixture. It has been proven in [20] that by increasing the amount of gum arabic admixture, and at the same time lowering W/C, the strength of concrete increased. On the other hand, other studies have usually used admixtures in amounts not exceeding 1%. In this case, this study confirms these observations, as a 1% admixture of gum arabic also lowered the compressive strength. In another work [23], after the modification of lime mortar with 3% admixture of sticky rice polysaccharides, there was a significant improvement in strength, which is also analogous to the results of this study. The decrease in strength after adding the polysaccharides contained in gums was also demonstrated in other studies on the modification of concretes and mortars based on Portland cement [20,21,22,48,49,50]. In turn, Elinwa et al. [51] proved that the compressive strengths of the concrete modified by gum arabic increased along with the dosage of admixture, and that the dosage range of 0.50–0.75% is adequate for use. When analyzing the results, it is not possible to observe the dependence, typical for building materials, between the porosity and the mechanical strength of the material. Usually, as the total porosity of the materials like mortars or concretes increases, their compressive and flexural strength decreases [52,53,54,55,56,57]. It is often a linear or close to linear relationship. In these studies, the conclusions are opposite. The mixture with the highest porosity has the greatest compressive strength. The size of the pores also affects the strength properties of building materials [58]. Scientific research has shown that despite the equal total porosity of the tested cement pastes and mortars, higher strength was characterized by those with smaller pores [54,59,60]. On the other hand, Lanes says that an increase in porosity can help enhance the strength of lime-based mortars due to the greater ability to carbonate [61]. 

A phenolphthalein test was performed to check the carbonation progress. This test was used in the investigation of various building materials (mortars, concretes, lime-based composites) [62,63,64]. The test is a comparative method, but to verify the exact amount of calcium carbonate in the sample, other analyses would have to be performed. The authors plan to thoroughly investigate the influence of gum arabic admixture on the carbonation and chemical processes occurring in the paste, as the strength results indicate that significant changes had taken place in the matrix.

The purple color in the photos (Figure 11) shows that phenolphthalein reacted with the un-carbonated region. The maximum depth of carbonation is 3.8–4.0 mm in the reference sample and the samples with 3% and 5% gum arabic. In the case of the sample with an admixture of 1%, the maximum depth is smaller and amounts to 3 mm; however, the breakthrough of samples from this recipe was the most irregular which could have influenced the reading. The most marked breakthrough and area of calcium carbonate occurred in the case of the LM-3GA formulation. The highest homogeneity of the samples could have contributed to the achievement of the highest bending strength. An interesting observation is the fact that the phenolphthalein reaction rates differ depending on the amount of gum arabic admixture. The admixture in the amount of 3% and 5% in the form of a solution introduced into the binder mixture thoroughly covered the lime and metakaolin particles, thus delaying the moment of direct contact of phenolphthalein with the lime particle. The test shows the effectiveness of the contact of lime and metakaolin particles with the gum arabic film, confirmed by the obtained strength parameters.

### 4.2. Hemp-Lime Concrete

#### 4.2.1. Mass Absorptivity

The maximal mass absorptivity of hemp-lime composites ranges from 86.6% to 96.7%. The results are comparable with the data in the literature [28,43]. The admixture of gum arabic decreased water absorption. As its content increases, the water absorption decreases. The admixture in the amount of 5% reduced the water absorption after 6 days by approximately 10% compared with the reference composite. In the case of admixtures in the amount of 1%, the effect is minimal and positive only after 1 day of saturation. The increase in the porosity of the binder with the increase in the gum arabic content did not increase the water absorption. The biopolymer dissolved in water covered the surface and probably filled some of the pores of the hemp shives, thus limiting water absorption. The concretes based on hemp shives are characterized by high water absorption in the first seconds after immersion in water [28,43]. The tested composites showed mass absorptivity in the range of 36.9–47% after 5 s of soaking in water, which is about 43–46% of the total amount of water that the composites are able to absorb.

#### 4.2.2. Compressive Strength and Young’s Modulus

**Figure 17:**
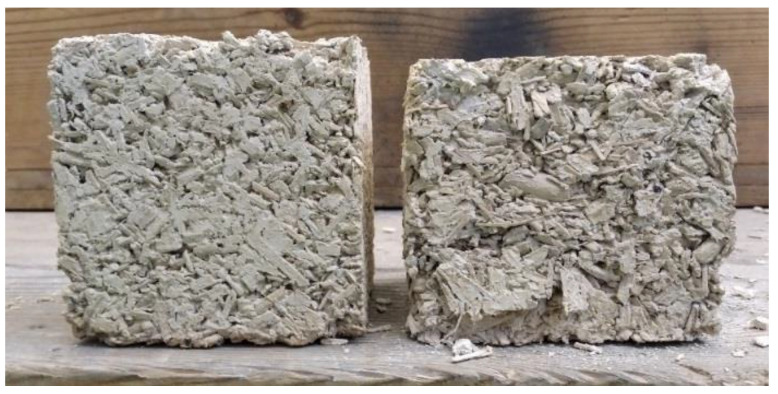
The form of destruction of composite specimen: Original sample (**left**), damaged sample—15% deformation (**right**).

The behavior of the samples under increasing axial load is comparable regardless of the recipe (Figure 13). They differ in the level of stresses, but it is not possible to identify the maximum, destructive stress in each case. This is a typical behavior of composite shives-based samples of varying length and loaded in the direction of mixture compaction [30,41,43,65,66]. Overall, the curves within one recipe are similar. There are no significant deviations in the behavior of the samples under increasing load. In the first phase of loading, the samples show quasi-elastic properties, the curve is linear, and the stresses increase rapidly, with a small increase in strains. In this phase, the main role is played by the binder and the adhesion strength of the binder to the shives. The graphs in the individual mixtures differ in the slope of the line in this phase. The higher the gum arabic content, the greater the degree of slope, and—therefore—the greater the stiffness of the material. Only the chart showing the mixture with 1% gum arabic shows a greater flattening than the reference composite. In the second phase, the charts are flattened. Stress grows slowly, while the strain increase is noticeable. After the binder is destroyed, the shives are compressed and the empty spaces between the shives are reduced. The loading was finished until the sample reached a deformation equal to 15%. A similar measuring range was adopted in [43,66]. With such deformation, the composite no longer fulfills its function because it is visually damaged, and is possible to be crushed in one’s hands. Figure 17 shows the form of destruction of composite specimen. The sample subjected to axial compression deforms in the vertical direction (its height decreases), but the typical damage associated with concrete samples based on Portland cement is not noticeable.

The immersion of the samples in water for 48 h significantly decreased the material’s resistance to increasing axial load (Figure 14). Drying the samples under natural conditions resulted in a new increase in strength, but the obtained values were lower than in the case of untreated samples. The smallest differences in the course of the stress–strain relationship of the original samples and dried samples can be noticed in the reference recipe without the addition of gum arabic. The greatest differences, on the other hand, are in the case of a sample containing an admixture of 5%. Hence, despite the fact that the admixture of gum arabic limited the water absorption, the water caused more damage than in the case of the reference samples. Some amount of gum arabic may have dissolved in water during immersion and could have been washed out of the composite structure. In this way, the bond strength of the binder with shives was weakened. In the reference mixture, the binder was only a mixture of lime and metakaolin, and as a result of the pozzolanic reaction, the binder was not destroyed by the influence of water.

Although it is not possible to read the maximum stress and calculate the compressive strength, it is possible to read the stress for a selected strain level. Elfordy et al. [65] determined the compressive strength at the onset of inelastic strain from the stress–strain curve. The same procedure was adopted in [30]. In other studies [43], for comparison purposes, the stresses were read for a deformation equal to 3.3%. The behavior of the samples was quasi-elastic within this range of deformations. In this study, as in [30,65], the point at which the stress–strain curve ceases to be linear was used to determine the compressive strength. The results are presented in Table 6.

The use of gum arabic in the amount of 3% and 5% significantly improved the strength of hemp concretes. To a minimal extent, this effect may also be due to the higher bulk density of these composites compared with HL (Table 5). According to the literature, the strength of composites increases with the increase in their density [57,65]. The admixture of gum arabic in the amount of 3 and 5% liquefied the binder, therefore the lime-hemp mixture was more susceptible to compaction. After soaking them with water, their strength drops to a similar level regardless of the amount of gum arabic, but the higher the admixture content, the greater the decrease. In the case of concretes modified with gum arabic, water caused an irreversible reduction in strength. The gum could partially dissolve, weakening the bond strength of the binder with the shives. Walker et al. [30] tested the strength of hemp-lime composites with the same binder-to-filler ratio (2:1) and a binder also containing metakaolin but in greater amount (20%). In the tests, they showed the compressive strength of composites was equal to 0.34 MPa. This is a similar result to that obtained in the present study for HL-0GA. On the other hand, in other studies [65], composites with a similar weight share of shives, but based on a commercial binder (BCB Tradical 70), were characterized by strength of the range of 0.18–0.60 MPa depending on the degree of compaction (404–485 kg/m^3^).

The average values of Young’s modulus of non-soaked hemp-lime composites are in the range of 6.3–12.5 MPa (Figure 15). The stiffness of the composites increases with the amount of gum arabic admixture; however, as in the case of the strength of pastes and composites, the admixture in the amount of 1% reduced the Young’s modulus value. Benfratello et al. [32] showed that the composites of similar density, but based mainly on hydraulic lime, had a modulus in the range of 16.3–20 MPa. The size of the shives also affects the stiffness of the composite. It has been proven in the literature [43] that the composites containing longer shives showed lower stiffness. In the cited studies [32], finer shives were used than in the present study. In other studies [65], the composites in the density range of 400–500 kg/m^3^ were characterized by Young′s modulus with values of 9–35 MPa. However, the binder contained 70% hydrated lime, 15% pozzolana and 15% hydraulic material.

Soaking the samples in water for 2 days significantly lowered the Young’s modulus values—by more than a half, with the exception of HL-1GA. After drying under natural conditions, the stiffness of the material returns to values close to the original values. The greatest difference can be seen in the samples with the addition of 5% gum arabic (Figure 15).

## 5. Conclusions

On the basis of the obtained results, it is possible to draw the following conclusions:

- Total porosity as well as average pore diameter increase along with the gum arabic content. Hence, the specific pore surface was substantially reduced.
- Regardless of the applied amount, the admixture significantly raised the number of pores with a diameter greater than 0.7 µm, in comparison with the reference paste.
- The 3% and 5% admixtures increased the flexural strength roughly by a factor of 3.
- The gum arabic addition to the lime-metakaolin mixture amounting to 3% and 5% enhanced the compressive strength of the paste by roughly 25% and 60%, respectively.
- The admixture of gum arabic increased the porosity of the paste without compromising the strength properties. The pastes characterized by the greatest porosity also exhibited the greatest compressive strength.
- The admixture of gum arabic decreased the water absorption of hemp-lime concrete. As the content increases, the water absorption decreases.
- The admixture of gum arabic in the amount of 3% and 5% improved the compressive strength of the hemp-lime composite by 53% and 92%, respectively.
- After immersing the hemp-lime composite samples in water, their compressive strength decreased by 45–63%. The re-dried samples have a compressive strength 26–36% lower in relation to the original samples. The higher the gum arabic content, the greater the decrease.

The obtained results are promising. The authors intend to investigate the chemical phenomena occurring in the modified paste, as well as the impact of the modification on the paste and hemp concrete durability (including frost resistance, salt resistance) and on the moisture properties.

## Figures and Tables

**Table 1:** Components of the tested pastes.

Recipe Symbol	Components		
	Binder	Gum Arabic/Binder Ratio	Water/Binder Ratio
LM-0GA	Hydrated lime 90% Metakaolin 10%	0	0.65
LM-1GA		0.01	
LM-3GA		0.03	
LM-5GA		0.05	

**Table 2:** Chemical composition of tested mixtures of paste.

Constituents	LM-0GA	LM-1GA	LM-3GA	LM-5GA
	Composition (g per 100 g)			
MgO	0.34	0.27	0.30	0.27
Al_2_O_3_	3.00	2.73	2.97	2.76
SiO_2_	5.42	5.37	5.14	4.86
SO_3_	0.19	0.20	0.20	0.21
K_2_O	-	0.06	0.11	0.07
CaO	90.33	89.61	87.52	86.10
TiO_2_	0.21	0.24	0.24	0.22
MnO	0.02	0.02	0.02	0.02
Fe_2_O_3_	0.49	0.50	0.50	0.48
ZnO	0.01	0.01	0.01	0.01

**Table 3:** Components of the tested hemp-lime concretes.

Recipe Symbol	Components		
	Binder	Hemp Shives/Binder Ratio (by Weight)	Water/Binder Ratio (by Weight)
HL-0GA	LM-0GA	1:2	1.35
HL-1GA	LM-1GA		1.375
HL-3GA	LM-3GA		1.29
HL-5GA	LM-5GA		1.24

**Table 4:** The average results of the paste tests performed by means of mercury porosimetry.

Parameter	Unit	LM-0GA	LM-1GA	LM-3GA	LM-5GA
Total pore surface	m^2^/g	13.70	11.36	11.21	11.28
Average pore diameter	nm	120.90	149.99	161.86	168.43
Total pore volume	ml/g	0.41	0.43	0.45	0.47
Total porosity	%	49.50	52.23	53.36	53.94
Density	g/mL	2.37	2.31	2.27	2.23
Bulk density	g/mL	1.20	1.17	1.12	1.10

**Table 5:** Bulk density of hemp concretes (average values) and the standard deviation.

Parameter	HL-0GA	HL-1GA	HL-3GA	HL-5GA
Bulk density [kg/m^3^]	445.4	442.4	446.7	451.1
SD [kg/m^3^]	±10.9	±9.6	±11.0	±6.6

**Table 6:** Average values of the compressive strength of composites.

Type of the Samples	Percentage of Gum Arabic/Average Compressive Strength [MPa]			
	0 GA	1 GA	3 GA	5 GA
HL	0.38	0.39	0.58	0.73
HL soaked	0.21	0.17	0.23	0.27
HL dried	0.41	0.25	0.43	0.49

## Data Availability

Data is contained within the article.
